# Implications of Hypoxia in Breast Cancer Metastasis to Bone

**DOI:** 10.3390/ijms17101669

**Published:** 2016-09-30

**Authors:** Daniele M. Gilkes

**Affiliations:** 1 Department of Oncology, The Johns Hopkins University School of Medicine, The Sidney Kimmel Comprehensive Cancer Center, Baltimore, MD 21231, USA; dgilkes1@jhu.edu; Tel.: +1-410-955-7371; Fax: +1-410-614-4073; 2 Department of Chemical and Biomolecular Engineering, The Johns Hopkins University, Baltimore, MD 21218, USA

**Keywords:** hypoxia, breast cancer, metastasis, bone, hypoxia-inducible factors (HIFs), invasion, migration

## Abstract

Most solid tumors contain regions of hypoxia in which increased cell proliferation promotes increased oxygen consumption and the condition is further exacerbated as cancer cells become localized far from a functional blood vessel, further decreasing the oxygen supply. An important mechanism that promotes cell adaptation to hypoxic conditions is the expression of hypoxia-inducible factors (HIFs). Hypoxia-inducible factors transcriptionally regulate many genes involved in the invasion and metastasis of breast cancer cells. Patients, whose primary tumor biopsies show high HIF expression levels, have a greater risk of metastasis. The current review will highlight the potential role of hypoxia in breast cancer metastasis to the bone by considering the regulation of many steps in the metastatic process that include invasion, migration, margination and extravasation, as well as homing signals and regulation of the bone microenvironment.

## 1. Breast Cancer Metastasis

Breast cancer is the most commonly diagnosed cancer among women in the world. Upon diagnosis, patients are classified into three main groups for treatment: (1) the hormonal receptor positive group (HR positive) characterized by estrogen receptor (ER) and/or progesterone receptor (PR) expression; (2) the human epidermal growth factor receptor 2 (HER2) amplified group, and; (3) the triple-negative breast cancer (TNBC) group, lacking expression of ER, PR and HER2. Approximately 6% of breast cancer patients have metastatic disease at diagnosis [1]. Over 90% of breast cancer patient deaths are due to metastasis, reflecting the poor response of patients with metastatic disease to currently available therapies. Bone is the most common site of metastatic lesion arising from breast cancer [2,3,4]. Other sites of breast cancer metastasis include lung, liver, and brain [2].

There are currently no methods for predicting whether a patient will experience metastatic disease progression or to predict the anatomical site of metastasis. Several research groups have utilized mouse models of site-specific metastasis coupled with “omics” analyses of tumor and metastatic tissue to develop gene expression signatures to predict bone [5,6,7,8,9,10], lung [11], and brain [12] metastasis. Taken together, the studies suggest that a distinct set of gene products may be required for the successful colonization of different organ types. Other studies have attempted to correlate organ-specific metastasis patterns with five major intrinsic molecular subtypes of breast cancer [3]: the luminal A and luminal B groups (characterized by the expression of luminal/epithelial markers); the HER2neu group (overexpressing the ERBB2 oncogene); the normal-like (closest to the molecular profile of a normal mammary gland); and the basal-like group (high expression of myoepithelial/mesenchymal markers) [13]. The results suggest that patients with basal-like tumors have higher rates of brain, lung, and distant lymph node metastases but a significantly lower rate of bone metastases [3,14] as compared to other molecular subtypes. Anatomical considerations for site-specific metastasis include blood flow patterns from the primary tumor to the metastatic organ, as well as the homing ability of cancer cells. Homing to specific organs is likely regulated by chemoattractant factors, as well as adhesion molecule expression in the target organ and counter receptor expression on the surface of cancer cells.

Breast cancer that metastasizes to bone results in an imbalance of normal bone remodeling caused by altered ratios of osteoclast-mediated bone resorption and osteoblast-mediated bone formation [15]. Bone metastases are classified on the basis of radiographic appearance as either osteolytic or osteoblastic. Breast cancer is more frequently associated with osteolytic type metastatic lesions [16]. The current standard of care for patients with bone loss due to osteolytic bone metastases includes anti-resorptive therapy aimed at reducing adverse skeletal-related events [16].

## 2. Hypoxia and Breast Cancer

Intratumoral hypoxia has been identified as an adverse indicator for patient prognosis independent of clinical stage at diagnosis [17,18]. Intratumoral hypoxia within a solid tumor occurs as cells proliferate and have an increase in the demand for O_2_. O_2_ availability is decreased due to structurally and functionally abnormal vessels that form within solid tumors [19]. Data describing the pretreatment oxygenation status of solid tumors show that the mean partial pressure of oxygen (*P*O_2_) in breast tumors ranges from 2.5 to 28 mm Hg, with a median value of 10 mm Hg, as compared to 65 mm Hg in normal human breast tissue [20]. *P*O_2_ values of less than 10 mm Hg have been associated with an increased risk of metastasis and mortality [18]. Given these dramatic findings, identification of key alterations that occur under hypoxic conditions is critical to elucidating mechanisms that promote metastasis.

Cancer cells respond to decreased oxygen availability by increasing the activity of the hypoxia-inducible factors, HIF-1 and HIF-2 [21]. HIF-1 functions as a heterodimeric protein composed of an O_2_-regulated HIF-1α subunit and a constitutively expressed HIF-1β subunit [22]. Under physiological oxygen concentrations, the HIF-1α subunit is hydroxylated on proline residue 564 and/or 402 causing the von Hippel-Lindau (VHL) E3-ubiquitin ligase complex to direct HIF-1α to the proteasome for degradation. Three oxygen-dependent prolyl hydroxylase enzymes (PHD1 (prolyl hydroxylase domain 1), PHD2 and PHD3) control the abundance of HIF proteins. Under hypoxic conditions, prolyl hydroxylation is decreased, resulting in HIF-1α accumulation and dimerization with HIF-1β [23]. The HIF-1 heterodimer binds to the consensus DNA sequence 5′-RCGTG-3′ which is present within hypoxia response elements and, along with coactivator proteins such as p300, leads to the transcriptional activation of HIF-1 target genes. HIF-2α is regulated in a similar manner and binds with HIF-1β to form the HIF-2 heterodimer. While HIF-1α and HIF-2α share a high degree of sequence similarity, HIF-2 causes the transactivation of some but not all of the genes activated by HIF-1 [24]. HIF target genes include many metastasis-related gene products [25] which may provide the mechanistic clues that support studies over the past decade that demonstrate a role for HIF-1α in adverse outcomes in breast cancer patients [26,27,28,29,30,31,32]. Regardless of lymph node involvement in breast cancer, survival outcomes are significantly decreased in patients with the highest HIF-1α levels in their diagnostic breast cancer biopsies [33,34].

Clinical findings on the importance of hypoxia in solid tumors have led to a large body of work aimed at characterizing the role of HIFs in experimental cancers. For example, breast cancers arising in conditional-knockout mice lacking HIF-1α expression in mammary epithelial cells showed significantly reduced lung metastasis compared to breast cancers arising in wild type mice [35]. In orthotopic transplants of human breast cancer cells injected into the mammary fat pad of immunodeficient mice, HIF-1 was also shown to be essential for the hematogenous metastasis of breast cancer cells to the lung [36,37]. Identifying the hypoxia-related events that could potentiate metastasis is an active field of investigation [38,39]. Several recent reviews suggest the potential for hypoxia to promote bone metastasis [40,41]. The current review will highlight studies of hypoxia-inducible gene products that may have relevance in the development of bone metastasis.

## 3. Hypoxia and the Bone Metastatic Cascade

Cancer cells spread via blood or lymphatic vessels which requires an invasion of adjacent tissue for efficient access to the blood stream. In order to migrate and invade the surrounding tissue, a cancer cell must decrease its cell–cell interactions, degrade the extracellular matrix (ECM), and then enter a blood or lymphatic vessel by a process known as intravasation. The cancer cell must survive within the circulation, and for bone metastasis to occur, cancer cells must adhere to capillaries within the bone marrow, extravasate and arrive “at” and survive “in” the bone marrow space. Many studies suggest that the metastatic environment itself must also be primed to provide a hospitable niche in order for cancer cells to survive in a distant organ [42]. The question of when, where and how the metastatic phenotype of cancer cells arises during the course of tumor progression is still unknown? Metastatic properties may arise very early within the evolution of the primary tumor (even prior to invasion). On the other hand, metastatic properties may be acquired very late as cells adapt to the microenvironment at the metastatic site [43]. Therefore, the potential exists that hypoxic signals at both the primary, as well as in the secondary site, can play a role in promoting cell growth in distant organs. In bone, for example, disseminated tumor cells could, in fact, experience hypoxic conditions in the bone marrow which is known to contain severe oxygen gradients [44].

In a study of 83 breast cancer patients with neither lymph node metastases nor overt distant metastases, high HIF-1α protein levels in primary tumor tissue correlated with the presence of cancer cells in bone marrow aspirates [45]. The presence of these early metastatic cells in the bone marrow predicts a postoperative occurrence of overt metastases in bone and other organs, suggesting a role for HIF-1α in the early phases of metastasis. The role of HIF-1α in the later stages of metastasis, such as bone colonization by breast cancer cells, has been tested in mouse models using MDA-MB-231 cells expressing a constitutively active or dominant negative form of HIF-1α [46]. MDA-MB-231 subclones were injected in the left ventricle of immunodeficient mice and allowed to form colonies within the bone. The tumor area and blood vessel density in long bone sections was significantly decreased in cells with reduced HIF-1 expression levels [46]. HIF-1α knockdown by shRNA also reduced the radiographic area of osteolytic lesions, decreased vessel density in bone metastases, and increased survival time in a second study [47]. Exposure of C3H10T1/2 mouse embryonic fibroblasts and mouse primary calvarial osteoblasts to hypoxia or constitutively active HIF-1α inhibited their differentiation and promoted osteoclastogenesis [46].

## 4. Hypoxia, Epithelial-to-Mesenchymal Transition (EMT), and Motility

**Figure 1:**
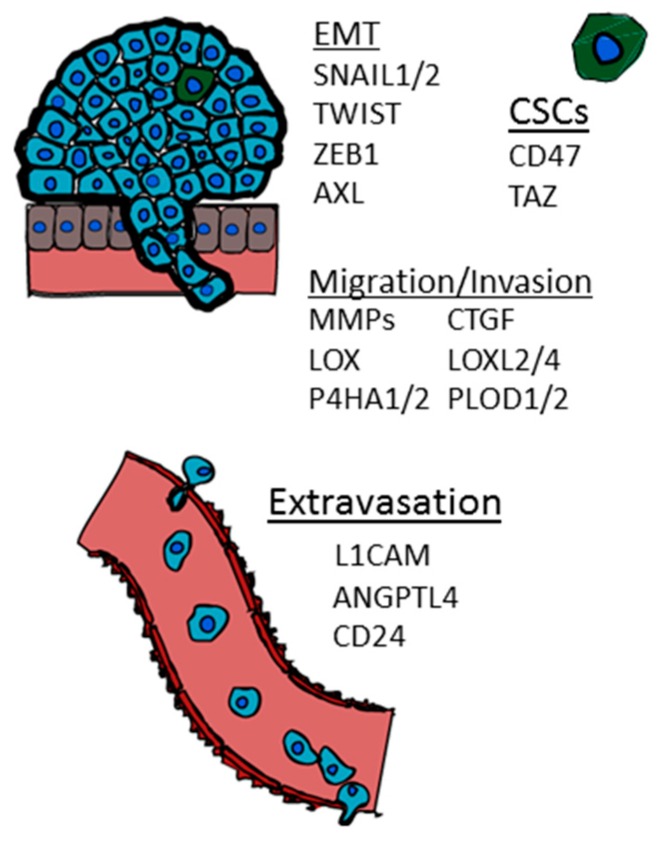
Hypoxia inducible genes that play a role in the epithelial to mesenchymal transition (EMT), cancer stem cell phenotype (CSC), migration, invasion, and extravasation.

The epithelial to mesenchymal transition (EMT) is a process by which epithelial cells lose their polarity and transition to a mesenchymal cell phenotype [48]. Hypoxia-inducible genes associated with EMT have been implicated in a wide range of cancers [48]. Genes including *SNAIL1*, *SLUG* (*SNAIL2*) and *TWIST* down-regulate E-cadherin expression, a protein which is important for adherens junctions and critical for epithelial cell–cell adhesion and tissue architecture (Figure 1). Loss or reduction of E-cadherin expression is frequently observed at the invasive front of advanced-stage human carcinomas [49]. In breast cancer cells, several studies have shown that hypoxia leads to an increase in the expression of two transcriptional repressors of E-cadherin, SNAIL1 and SLUG, by modulating the NOTCH1 signaling pathway [50,51,52], and HIF-1 transactivation of both the SNAIL1 and SLUG promoters has been demonstrated [53,54]. E-cadherin expression has also been shown to increase through a HIF-1-dependent regulation of PPARγ in the MDA-MB-231 bone metastatic derivative (BO 1833) cell line [55].

HIF-1α also directly regulates the expression of TWIST in breast cancer cells [56] (Figure 1). During development, TWIST promotes gastrulation and mesoderm specification. In breast cancer, TWIST expression results in the loss of E-cadherin-mediated cell–cell adhesion, upregulation of mesenchymal markers, and induction of cell motility [57]. Hypoxia or overexpression of HIF-1α induces a metastastic phenotype in otherwise non-metastatic breast cancer cells via a TWIST-dependent mechanism in vivo; TWIST short-interfering RNA (siRNA) can reverse this effect [56]. In addition to TWIST, AXL, a receptor tyrosine kinase, was recently identified as a novel HIF target gene capable of promoting EMT, invasion, and metastasis in both VHL-deficient as well as hypoxic cancer cells (Figure 1) [58]. Recent literature also supports a role for AXL in promoting metastasis in many tumor types, including breast [59], ovarian [60], and lung [61].

ZEB1, a transcriptional repressor, is hypothesized to have the potential to initiate bone metastasis by inducing osteoclast formation in an MMP-1 dependent manner [62]. ZEB1 expression directly promotes BMP-inhibitor gene transcription and indirectly suppresses BMP-inhibitor reduction via miR-200 family members. As a result, ZEB1 expression leads to BMP-inhibitor mediated osteoclast differentiation [63]. In colon cancer cells, HIF-1α directly binds to a hypoxia response element (HRE) in the proximal promoter of ZEB1 causing an increase in the transactivation and expression of ZEB1. In addition, inhibition of ZEB1 abolished HIF-1α-induced EMT and cell invasion [64].

## 5. HIF Regulation of Invasion and Intravasation

Cells at the invasive front of solid tumors are frequently found to have high expression levels of HIF-1α [59]. Invading tumor cells are thought to degrade the basement membrane that surrounds them by activating matrix metalloproteinases (MMPs), endopeptidases that degrade ECM components. MMP-2 and MMP-9 degrade type IV collagen, a major component of the basement membrane. Hypoxia induces increased expression and activity of MMP-2 and MMP-9 through a HIF-1-dependent process (Figure 1) [60,61], and increased levels of MMP-2 in breast cancer biopsies were associated with poor prognosis [62]. In addition to secreted MMPs, which are activated by extracellular proteolytic cleavage, hypoxia also induces the membrane bound MT1-MMP in cancer cells by direct binding of HIF-2 to an HRE in the MMP14 gene locus [63].

Although degradation of ECM by proteases has been established as an important mechanism for tumor cell invasion, recent evidence shows that collagen, in particular type I collagen, provides a roadway for cell migration during invasion [65,66]. Using mouse models that recapitulate the histological progression of human breast cancer, mammary tumors exhibit a localized increase in collagen deposition, which occurs early in tumor formation [64,65]. As tumor size increases, collagen fibers straighten, bundle, and align [66]. Several groups have observed that tumor cells preferentially invade along aligned collagen fibers [66,67,68]. Furthermore, the pattern and extent of collagen alignment has a prognostic significance in breast cancer [69]. Together this suggests that ECM matrix remodeling is highly dynamic. Cells must both degrade and reform an extracellular matrix scaffold to support their migration within the tumor mass.

HIF-1α promotes collagen biogenesis and alignment in breast tumors by the transcriptional activation of collagen prolyl (P4HA1 and P4HA2) and collagen lysyl (PLOD1 and PLOD2) hydroxylases (Figure 1) [70,71,72] as well as lysyl oxidase family members (LOX, LOXL2, LOXL4) under hypoxia [73,74]. Proper hydroxylation is required for the folding of newly synthesized procollagen polypeptide chains into stable triple-helical molecules and is a requirement for subsequent secretion into the extracellular space [75,76]. The knockdown of either HIF-1α, P4HA1, or P4HA2 decreases tumor fibrosis and P4HA1 or P4HA2 knockdown completely abrogates the spontaneous metastasis of mammary fat pad-implanted human breast cancer cells to the lungs and lymph nodes of immunodeficient mice [72]. In contrast to the effects of P4HA1 or P4HA2 knockdown, the knockdown of PLOD2 expression does not suppress collagen deposition but instead compromises collagen crosslinking. PLOD2 knockdown significantly impairs the spontaneous metastasis of mammary fat pad-implanted breast cancer cells to mouse lungs and lymph nodes [70].

## 6. Hypoxia and the Cancer Stem Cell Phenotype

The cancer stem cell theory posits that rare cancer cells with infinite growth and variable potential exist within a primary tumor [77]. It is tempting to presume that these cells may also have a propensity or enhanced ability to metastasize. Hematopoietic stem cells (HSCs) that are localized within the bone marrow have a self-renewal ability and can differentiate to a number of cell lineages. Interestingly, HSCs are found in regions of the bone marrow with the lowest oxygen content [46], suggesting that hypoxia could play an essential role in stem cell fate. Interestingly, HSC differentiation occurs adjacent to osteoblasts located in the bone marrow cavity [78], suggesting that, similar to HSCs, CSCs may thrive and differentiate in a hypoxic bone microenvironment.

Hypoxia has been shown to induce a breast cancer stem cell phenotype in a HIF-dependent manner [79]. The stem cell phenotype is generally dictated by a specific gene expression pattern. In breast cancer studies, the co-activator TAZ (transcriptional activator with PDZ-binding motif) can promote the rare population of CSC to self-renew, endowing them with tumor-initiation abilities [80]. The role of TAZ in development is to regulate organ mass [81]. TAZ is phosphorylated by LATS1 or LATS2 which causes TAZ to be maintained in the cytosol, preventing its interaction with DNA-binding proteins of the TEAD (TEA DNA binding domain) family to activate transcription. TAZ mRNA and protein expression are induced by hypoxia in a HIF-1α-dependent but HIF-2α-independent manner (Figure 1) [82,83]. HIF-1α binds to an intron region within the WWTR1 gene. Luciferase reporter assays were utilized to identify a functional hypoxia response element in order to demonstrate that WWTR is a direct HIF-1 target gene. TAZ binding to the connective tissue growth factor (CTGF) promoter was increased under hypoxic conditions which led to a HIF-1α-dependent increase in CTGF mRNA levels. Taken together, the studies show that HIF-1 can promote TAZ expression leading to enhanced TAZ target gene activation in hypoxic breast cancer cells [82,83].

An analysis of circulating tumor cells isolated from the blood of patients with breast cancer revealed a population of metastasis-initiating cells (MICs) that express EPCAM, CD44, CD47 and MET. CD47 expressing cells were capable of initiating bone metastasis when injected into the femurs of immunodeficient mice [84]. HIF-1 activates the transcription of the CD47 gene under hypoxia. Reducing either HIF-1 or CD47 expression can cause an increase in bone-marrow derived macrophage phagocytosis of breast cancer cells. CD47 expression is enhanced in mammosphere cultures whereas inhibiting CD47 leads to cancer stem cell depletion in mammospheres [85]. TAZ and CD47 serve as two examples of the potential role of hypoxia in driving a stem cell phenotype (Figure 1).

## 7. Hypoxia and Extravasation

Extravasation involves modulation of tumor cell adhesion to the endothelium of blood vessels. HIF target genes that promote extravasation have recently been identified [37]. L1CAM, a protein involved in cell–cell adherence by homophilic interactions or by heterophilic interactions with integrins, neuropilin 1 and CD24, is transcriptionally activated by HIFs. Breast cancer cells exposed to hypoxia for 48 hours displayed an increase in adhesion to ECs. Abrogating the expression of HIF-1 or L1CAM significantly reduced cell adherence to ECs. MDA-MB-231 cells engineered to express HIF-1 levels below basal levels while overexpressing L1CAM were directly injected into the circulation system of mice which promoted enhanced extravasation compared to control MDA-MB-231 cells with basal expression of HIF-1 and L1CAM [37]. Hypoxia also induces the expression of the L1CAM-interacting protein CD24 [37,67,68]. HIF-1α overexpression led to increased CD24 mRNA and protein levels. Similar to L1CAM, attenuation of CD24 by shRNA expression reduced metastasis. CD24 overexpression in HIF-1α-knockdown cancer cells rescued this decrease while HIF-1α overexpression in CD24-knockdown cells did not. Although these studies were not performed in breast cancer cells, immunohistochemical expression of CD24 in early primary invasive breast cancers has been significantly associated with poor prognosis [69]. Taken together, this suggests a potential role for HIF-1 regulation in CD24 overexpression in breast cancer.

Angiopoietin-like 4 (ANGPTL4) was also found to be a hypoxia-induced and HIF-dependent gene product that promotes extravasation of breast cancer cells in in vivo models of lung metastasis [37,70]. TGF-β induction of ANGPTL4 expression in cancer cells disrupts vascular endothelial cell–cell junctions, increases the permeability of lung capillaries, and facilitates the *trans*-endothelial passage of tumor cells [71]. In vitro assays, using a conditioned medium from breast cancer cells, suggest increased extravasation is due to reduced EC–EC adherence. For example, conditioned media from breast cancer cells stably transfected with an ANGPTL4 expression vector inhibited EC–EC interactions as measured by transendothelial electrical resistance and breast cancer invasion assays. When breast cancer cells are injected via tail vein, HIF-knockdown cells overexpressing ANGPTL4 extravasated more readily to the lungs [37]. Lung metastasis from ANGPTL4-knockdown breast cancer cells following mammary fat pad implantation were also significantly reduced compared to mice bearing control tumors. Additional in vivo studies to assess vascular permeability and metastasis, performed using *ANGPTL4* knockout and wild-type mice injected with either control or ANGPTL4-knockdown tumors, confirmed that ANGPTL4 induces vascular leakiness and promotes lung metastasis in mice [72]. Clinically, ANGPTL4 is expressed at increased levels in the primary breast cancers of women with lung metastases [70]. The role of ANGPTL4 in the bone metastasis of breast cancer cells has yet to be determined. ANGPTL4 overexpression can cause up-regulation of bone morphogenetic protein 7 (BMP7) in in vitro studies [73] and has been implicated in osteoblast differentiation [74], suggesting the role of ANGPTL4 in bone metastasis should be explored. ANGPTL4 and L1CAM provide two important examples of HIF-induced proteins that have been shown to play a role in the extravasation of breast cancer cells (Figure 1).

## 8. Hypoxia and Homing/Priming of the Bone Microenvironment

**Figure 2:**
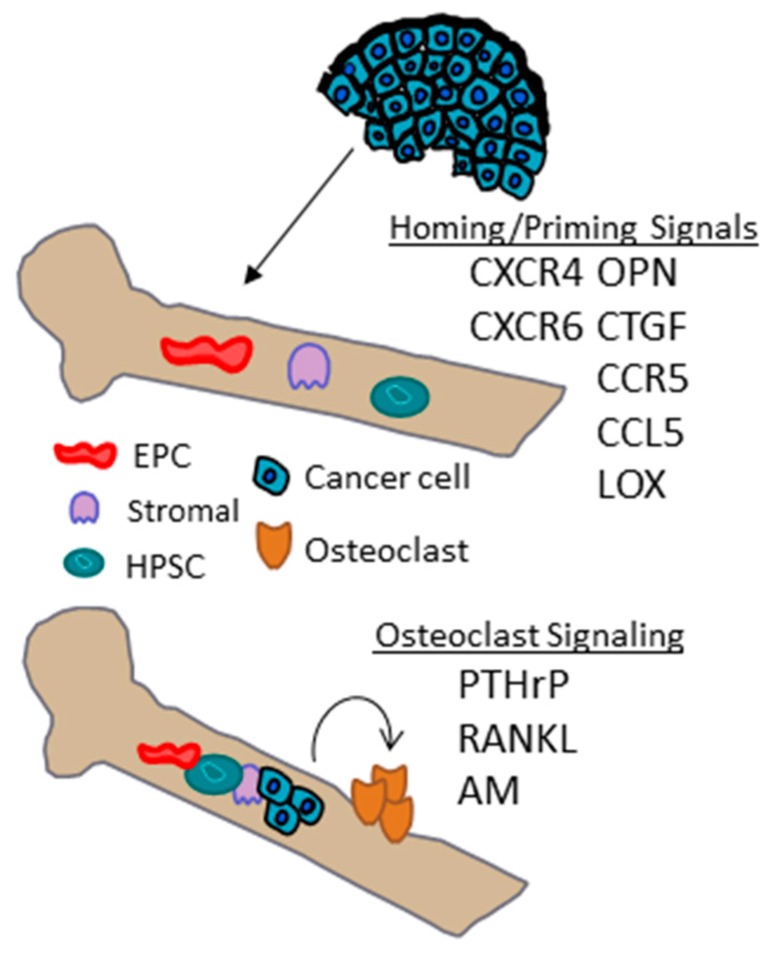
Hypoxia inducible genes that may play a role in bone homing and osteoclast signaling.

The homing of breast cancer cells to metastatic sites may also be governed by interactions between chemokine receptors on cancer cells and ligand secretion in target organs (Figure 2). The stromal-cell derived factor 1 (SDF-1)/chemokine receptor-4 (CXCR4) signaling complex may play an important role in bone metastasis. For example, SDF-1 is abundant in bone marrow stromal cells [75]. Expression of the genes encoding SDF-1 and CXCR4 are induced by hypoxia in a HIF-dependent manner in various cell types [76,77,78,79]. In vivo studies inhibiting CXCR4 expression by short interfering RNA or blocking its function with either neutralizing antibodies or synthetic peptides can inhibit lung metastasis in orthotopic models [80,81]. Additionally, SDF-1/CXCR4 signaling in ECs under hypoxic conditions leads to tube formation, adhesion of breast cancer cells to ECs and stimulates transendothelial migration in a HIF-dependent manner [82]. Breast cancer cell adhesion and migration through a normal human umbilical vein endothelial cell (HUVEC) monolayer is significantly reduced by inhibiting CXCR4 or treating ECs with an SDF-1 neutralizing antibody. Importantly, CXCR4 is a predictive marker for bone metastasis in breast cancer patients with visceral metastases [83]. Hypoxia-induced and HIF-dependent expression of additional chemokines and their ligands, such as CXCR6, CCR5, and, CCL5 have also recently been implicated in enhancing directed migration of breast cancer cells [84,85].

In addition to *CXCR4*, *CTGF* and osteopontin (*OPN*) are two genes that also favor homing and/or adherence of cancer cells to bone. Osteopontin has been shown to be hypoxia-inducible in glioblastoma cell lines [86]. Connective tissue growth factor is also a hypoxia-regulated protein that is induced in a HIF-1 dependent manner [87]. Hypoxia response elements identified upstream of the *CTGF* basal promoter enable a direct interaction of HIF-1, resulting in the increased transcription of CTGF mRNA. Cells deficient in HIF-1 were incapable of inducing CTGF in response to hypoxia [87].

In addition to the role of the LOX in premetastatic niche formation in the lung and collagen crosslinking in the primary tumor, a global quantitative analysis of the hypoxic secretome identified LOX as being significantly associated with bone-tropism and relapse. Stratifying patients based on mRNA levels of a hypoxia-inducible gene signature demonstrated an increase incidence in bone relapse among a subset of patients with ER negative breast cancer that express high levels of a hypoxia-inducible gene signature. In animal models, LOX expression leads to osteolytic lesion formation prior to the arrival of tumor cells [9]. LOX was found to regulate NFATc1-driven osteoclastogenesis. The osteoclastic lesions provide a niche for tumor cells to colonize and form bone metastases [9].

## 9. Hypoxia Regulates Osteoclasts in the Bone Microenvironment

Bone is a dynamic organ composed of many bone marrow-derived cell (BMDCs) types, including hematopoietic, mesenchymal, and endothelial cells and the bone microenvironment harbors hypoxic regions. Once cancer cells arrive in the bone marrow they must adhere to the bone matrix and promote osteoclast formation to establish bone metastases [88]; the hypoxic microenvironment in the bone may play a role in this process. For example, HIF-1α activation in response to hypoxic conditions can promote the recruitment of bone cell precursors, and also may have direct effects on osteoblast and osteoclast differentiation and activity [41,89]. A full discussion of the regulation of secreted proteins produced by cancer cells that may modulate the response of cells in bone microenvironment is beyond the scope of this review, but a discussion of three secreted-proteins that are induced by HIFs under hypoxia and that have been implicated in bone metastasis are highlighted as examples.

Cytokines produced by breast cancer cells act upon host cells of the bone microenvironment to promote osteoclast formation, allowing for excessive bone resorption. Interestingly, hypoxia induces parathyroid hormone related protein (PTHrP) secretion and gene expression in prostate, breast, and colon cancer cells [90]. PTHrP expression by breast cancer cells leads to osteolytic bone destruction [88]. PTHrP transcription is enhanced by HIF-2α which binds to hypoxia response elements in the PTHrP promoter region [90]. Since tumor cells can be exposed to hypoxia in the bone marrow [44], PTHrP secretion can be enhanced in the bone marrow microenvironment. PTHrP was found to be expressed in 60% of primary tumors analyzed but was not expressed in paired, normal breast tissue [91]. In a comparative study of primary breast cancers with metastases to the bone or soft tissue, 92% of bone metastasis were positive for PTHrP expression compared to only 17% in non-bone tissues [92,93], suggesting that PTHrP expression in the primary tumor could promote preferential localization and growth in bone.

The receptor activator of nuclear factor (NF)-κB ligand (RANKL) and its cognate receptor RANK are essential mediators of osteoclast function and survival [94]. Preclinical data established that inhibiting RANKL prevents tumor-induced osteoclastogenesis, protecting against bone destruction and inhibiting the progression of established bone metastases [94]. Hypoxia induces RANK and RANKL mRNA and protein in MDA-MB-231 and MCF-7 breast cancer cells in a HIF-1α dependent manner, and has also been shown to accelerate RANKL-mediated cell migration [95]. In bone metastasis, RANKL-positive stromal cells have been observed at the tumor/bone interface adjacent to osteoclasts but whether hypoxia could promote RANKL in stromal cells remains to be determined.

In addition to RANKL, hypoxia has also been shown to induce adrenomedullin (AM). Breast cancer cells, genetically altered to express a fivefold increase in the expression of AM, were inoculated into the left ventricle of immunodeficient mice, causing osteolytic bone metastases to develop more rapidly and enhanced cancer cell proliferation in the bone [96]. An X-ray analysis of bone showed increased osteolytic activity which was accompanied by an increased numbers of osteoclasts.

## 10. Additional Mechanisms of HIF Regulation

In addition to hypoxia, HIFα proteins are also regulated in response to various stress conditions. Many of these conditions result in downstream signaling mechanisms that culminate with the activation of protein kinases that directly or indirectly regulate HIFα levels. Several studies have shown that the PI3K/Akt pathway can induce HIFα stabilization [97,98,99,100] and coactivator recruitment [101]. The AKT pathway is only one example of HIF regulation by kinases. mTOR, p38, MAPK, and AMPK serve as additional examples [102]. HIFα regulation by phosphorylation is likely a cell type specific event that varies according to cell-type and signaling event.

Interestingly, hypoxia has also been shown to regulate the activity of some kinases and thus may result in a feedback loop. For example, hypoxia can enhance the PI3K/Akt pathway causing an increase in HIF-1α protein levels, whereas prolonged/chronic hypoxia increased GSK3β activity which led to decreased HIF-1α protein levels [103]. This effect is also likely to be cell-type specific as the MEK1 inhibitor PD98059 has been shown to have a suppressive effect in hypoxia-mediated HIF-1α transcriptional activity in Hep3B and HMEC-1 cells [104,105], whereas the same inhibitor was ineffective in fibroblasts exposed to hypoxia [106]. Whether any of these additional mechanisms plays a role in promoting metastasis specifically to bone remains to be determined.

## 11. Conclusions

The majority of studies focused on breast cancer metastasis in the literature have utilized lung as the model metastatic organ. The availability of mouse tumor models that recapitulate breast cancer metastasis to bone is clearly lacking. An even greater challenge lies in corroborating data obtained from mouse models in a human setting in which metastatic human tissue samples must be obtained. Although the role of hypoxia in organ-specific breast cancer metastasis is currently unknown, the studies highlighted above suggest that many hypoxia-inducible genes have the potential to play a role in promoting the metastasis of breast cancer to the bone. HIF-1α expression has been implicated as an independent predictor of a poor outcome for breast cancer patients, suggesting that HIF-1α levels in the diagnostic tumor biopsy could be used to identify patients that are at increased risk of developing metastasis. Hypoxia is present in over 90% of solid tumors. Therefore, potential findings in the breast cancer setting are likely to be relevant in other cancer types. Continued studies which examine specific mechanisms of the role of hypoxia in metastasis are clearly warranted and may likely lead to new and innovative therapeutic strategies to block metastasis.

## Abbreviations

HIF	hypoxia inducible factor
EMT	epithelial to mesenchymal transition
HR	hormonal receptor
ER	estrogen receptor
PR	progesterone receptor
HER2	human epidermal growth factor receptor 2
HRE	hypoxia response element
P4H	collagen prolyl hydroxylase
PLOD	collagen lysyl hydroxylase
LOX	lysyl oxidase
AM	Adrenomedullin
SDF-1	stromal-cell derived factor 1
CXCR4	chemokine receptor-4
BMDCs	bone marrow-derived cell
HSCs	hematopoietic stem cells
CSCs	cancer stem cell
TAZ	transcriptional activator with PDZ-binding motif
CTGF	connective tissue growth factor
ECs	endothelial cells
PTHrP	parathyroid hormone related protein
RANKL	receptor activator of nuclear factor kappa-B ligand
